# Case report on refractory epistaxis successfully treated with a novel taping therapy: A new approach

**DOI:** 10.1097/MD.0000000000050558

**Published:** 2026-09-04

**Authors:** Hwa Soo Hwang, Bong Hyo Lee

**Affiliations:** a Chims Saeng–Vit Korean Medicine Clinic, Seoul, South Korea; b Department of Acupuncture, Moxibustion, and Acupoint, College of Korean Medicine, Daegu Haany University, Gyeongsan-si, Gyeongsangbuk-do, South Korea.

**Keywords:** bio-photon, bleeding, nasal, painful response, quartz

## Abstract

**Rationale:**

Epistaxis is a very common nose disease that often requires hospital admission. Chemical therapies, surgery, and nasal packing have mostly been used for treatment; however, it remains prevalent, and noninvasive treatments using meridian and acupuncture theory have rarely been studied. Here we report two chronic cases that were successfully treated with novel taping therapy.

**Patient concerns:**

First, a 62-year-old male patient with right-side epistaxis, which appeared almost every morning and had lasted for6 months visited. Second, a 9-year-old female patient with both-side epistaxis visited. The symptom appeared 1 to 2 times a week and had lasted for 3 years. As present history, she had chronic fatigue, frequent eye blinking, and nasal congestion. She was a light eater and used to go to bed at midnight.

**Diagnoses:**

First, he had been treated for epistaxis, and the hospital had treated the patient with laser therapy; however, it was not effective. Second, she had visited the hospital approximately 20 times a month with epistaxis and had been given laser therapy.

**Interventions:**

At first, finger pressure was performed to examine if there was a painful response in the sternocleidomastoid muscle and the acupoints around the nasal sinus. At the painful response sites, we attached the medical tape. The effectiveness of this therapy was evaluated mainly using the visual analog scale.

**Outcomes:**

In case 1, every morning nose bleeding that had lasted 6 months was successfully treated with 12 sessions. During the treatment period, it recurred whenever the patient overworked. After final treatment, it did not recur until one month later. In case 2, epistaxis that had lasted for 3 years improved immediately after our taping at the initial visit, eventually being terminated with 5 sessions.

**Lessons:**

Attachment of the quartz Chimsband at the painful response sites of the sternocleidomastoid muscle and acupoints around the nose has been shown to be effective for refractory epistaxis. In conclusion, using quartz in a medical tape to reflect the bio-photon can be an effective strategy to treat chronic diseases, and the Chimsband using quartz is a novel taping therapy for refractory epistaxis.

## 1. Introduction

Epistaxis is a common nose disease with a prevalence of 60%.^[1]^ Usually Western medicine preferentially considers conservative treatment however, surgery can’t be avoided when the conventional treatment and chemical medications are ineffective, and 1.6 patients per 10,000 still show serious status that needs hospital admission.^[2]^

Recently, biomedical research has been developing in diverse ways, including 3D organoids and organ-on-chip, and in addition, the combination with gene editing, multi-omics profiling, and machine learning has been facilitating personalized medicine.^[3]^ Nevertheless, conventional treatment of Western medicine should better be carefully performed,^[4]^ and in this light, complementary and alternative medicine has been gaining interest.

Whereas, in the traditional medicine of Eastern Asia, such as Korea, treatment approaches used to be carried out on the functional aspect at first rather than on the structural aspect, and epistaxis has been understood to result from *Rising Heat*, which is associated with bulimia and/or fatigue after overwork. Also, it was described in ancient textbooks that methods of cooling blood and/or promoting blood circulation using nasal inhalation were usually performed.^[5]^

However, similarly with Western medicine, the mainstream treatments for epistaxis in Korean traditional medicine have been pharmacological treatments using herbal medications,^[6–9]^ and non-pharmacological treatments including acupuncture have been less reported. Furthermore, taping therapy for epistaxis has not been studied yet. Therefore, here we report for the first time 2 cases that were successfully treated with a novel taping therapy.

In Eastern Asian traditional medicine, meridian theory provides the fundamental foundation for non-pharmacological treatment such as acupuncture, and according to meridian theory, the local site of epistaxis belongs to the area of Yang Brightness (陽明).

The acupoints around the nasal cavity, such as LI20 of Hand Yang Brightness (手陽明), ST2, and ST3 of Foot Yang Brightness (足陽明) and the sternocleidomastoid muscle, serve as important sites for the treatment of nose diseases, and diverse therapies including acupuncture, massage, thermotherapy, and physical therapy using electromagnetic waves have been utilized.^[10,11]^

On the other hand, it is known in the field of physical treatment that if there is a disease or health problem, an electromagnetic wave, so-called bio-photon, appears in the place where inflammation and/or a painful response occurs and reactive oxygen is exhibited.^[12]^ Also, an injury current, a weak bioelectric current, appears, and using this injury current has been known to be effective for treatment or recovery.^[13]^ In our previous studies, we devised a novel medical tape (Chimsband) using silver and optic fiber to resolve the painful response at disease-related muscle trigger points (TPs) and acupoints, and the therapeutic effects were thought to be mediated via regulating the injury current, i.e., bioelectric current.^[14–17]^ Based on this, we developed the medical tape and used quartz instead of silver and optic fiber. Quartz, a medical ingredient described in the Dong-Ui-Bo-Gam (東醫寶鑑),^[18]^ has an outstanding ability of reflection and penetrability of light.^[19]^ This feature was thought to be beneficial for using the bio-photon which appears at the painful response site and therefore to be good at resolving the painful response. Therefore, we anticipated that this quartz Chimsband would be effective to treat a disease and examined whether chronic epistaxis could be improved with this tape.

## 2. Materials and methods

### 2.1. Materials

**Figure 1.**
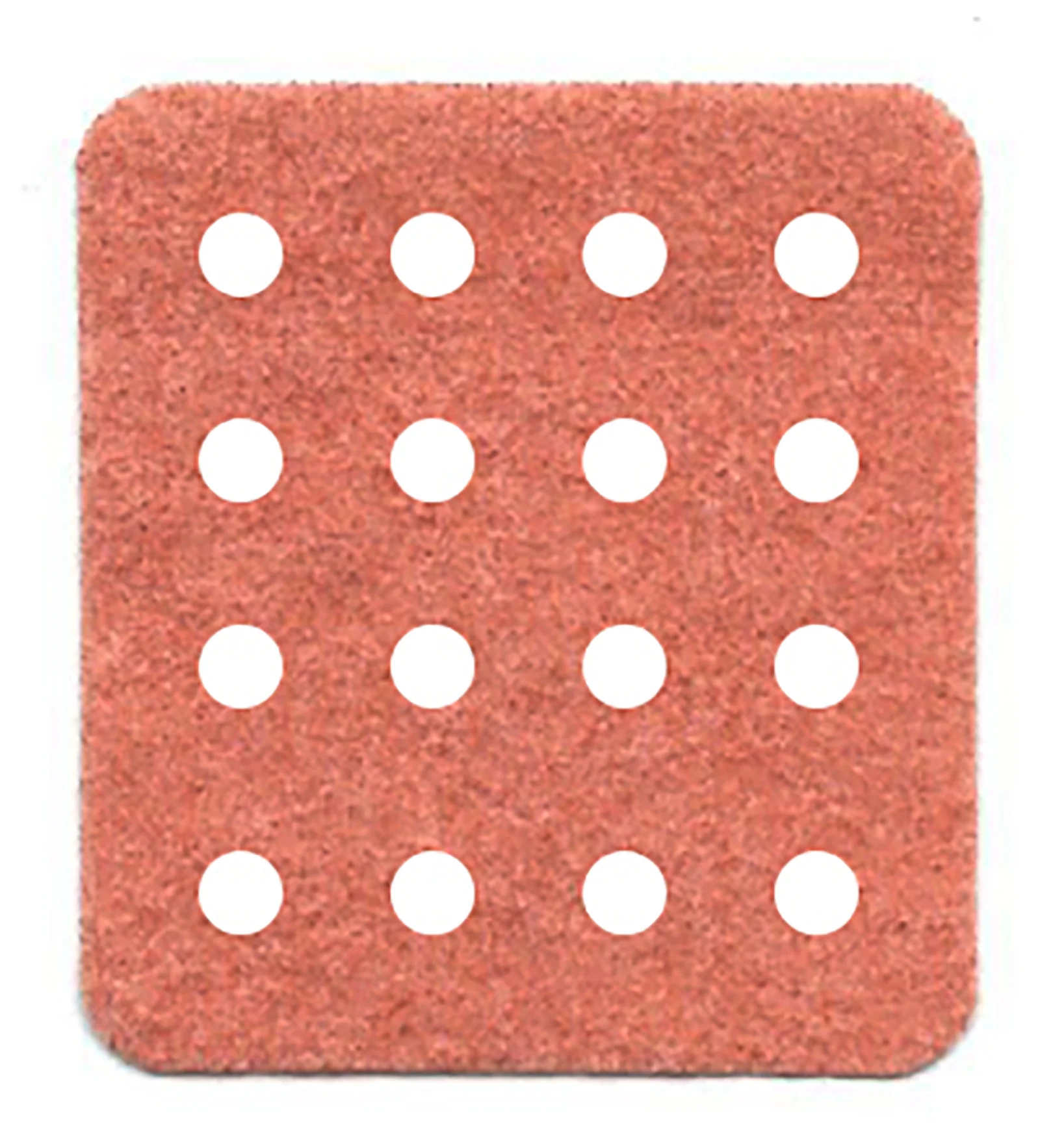
Appearance of the quartz Chimsband.

In this study, a novel medical tape (Quartz Chimsband; Chims Saeng-Vit Korean Medicine Clinic, Seoul, Korea) measuring 3 × 3.2 cm^2^. One piece of the tape has 16 quartz sites where quartz was applied under a precision process (Fig. 1). The diameter of the circle-shaped quartz site was 2 mm, the distance between quartz sites was 4 mm, and the size of the quartz particle was <300 nm. Quartz was used in this medical tape because it has an excellent ability to regulate biophotons, which naturally occur at related painful response sites under disease conditions. Quartz has been referred to as having high-level light permeability in the Dong-Ui-Bo-Gam. In this study, we used high-quality quartz that had been revealed to be safe through toxicity testing.

## 3. Methods

### 3.1. Finger examination

**Figure 2.**
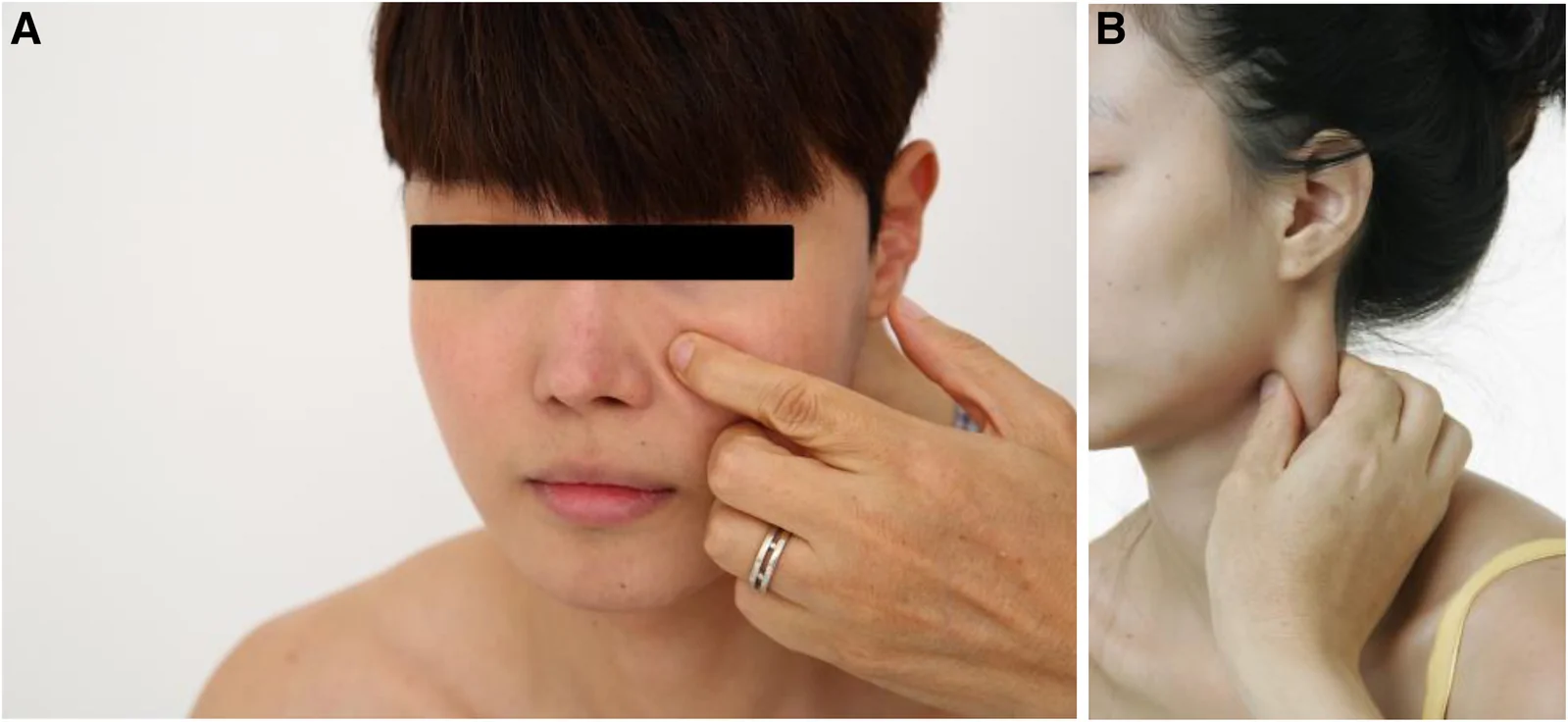
Finger pressure examination at the LI20 acupoint (A) and the sternocleidomastoid muscle (B).

Basically, a routine examination was carried out by pressing the acupoint perpendicularly with a finger at the LI20 and around (Fig. 2A) and grabbing the TP with fingers at the sternocleidomastoid muscle (Fig. 2B), and then checking if there was any painful or sensitive response.

### 3.2. Intervention

**Figure 3.**
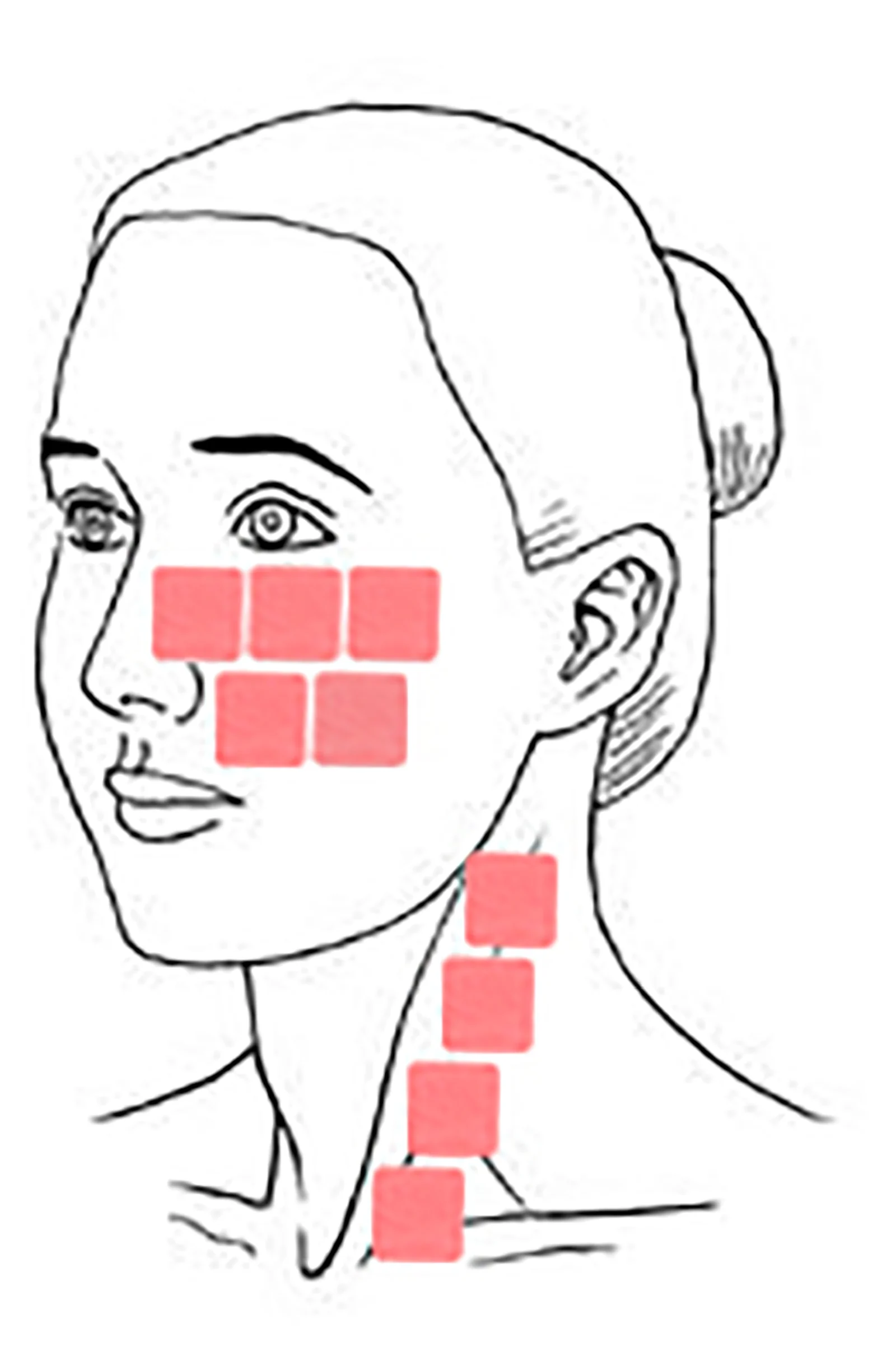
Schematic of the attachment of the quartz Chimsband around LI20 and the sternocleidomastoid muscle.

The quartz Chimsband was attached at the painful or sensitive response site (Fig. 3) based on the finger examination, and no other treatment was added.

### 3.3. Evaluation of effectiveness

The efficacy of the intervention was evaluated mainly by the subjective assessment of the patient (visual analogue scale, VAS), and the change of the accompanying symptoms was supplementally considered.

### 3.4. Ethic information

This study was performed in compliance with the general ethics that a case report should follow and under the intended consents of the patients. Also, this study was approved by the Institutional Review Board of Daegu Haany University (DHU2025-1-03).

## 4. Case presentation

### 4.1. Case 1

#### 4.1.1. History and examination

A 62-year-old male patient presented with right-side epistaxis which had occurred almost every morning and had lasted for 6 months. He had no special past or family history. The hospital had treated the patient with laser therapy; however, it was not effective.

When we examined around the LI20 and the sternocleidomastoid muscle on the right side, painful responses were shown.

#### 4.1.2. Treatment and progress

**Figure 4.**
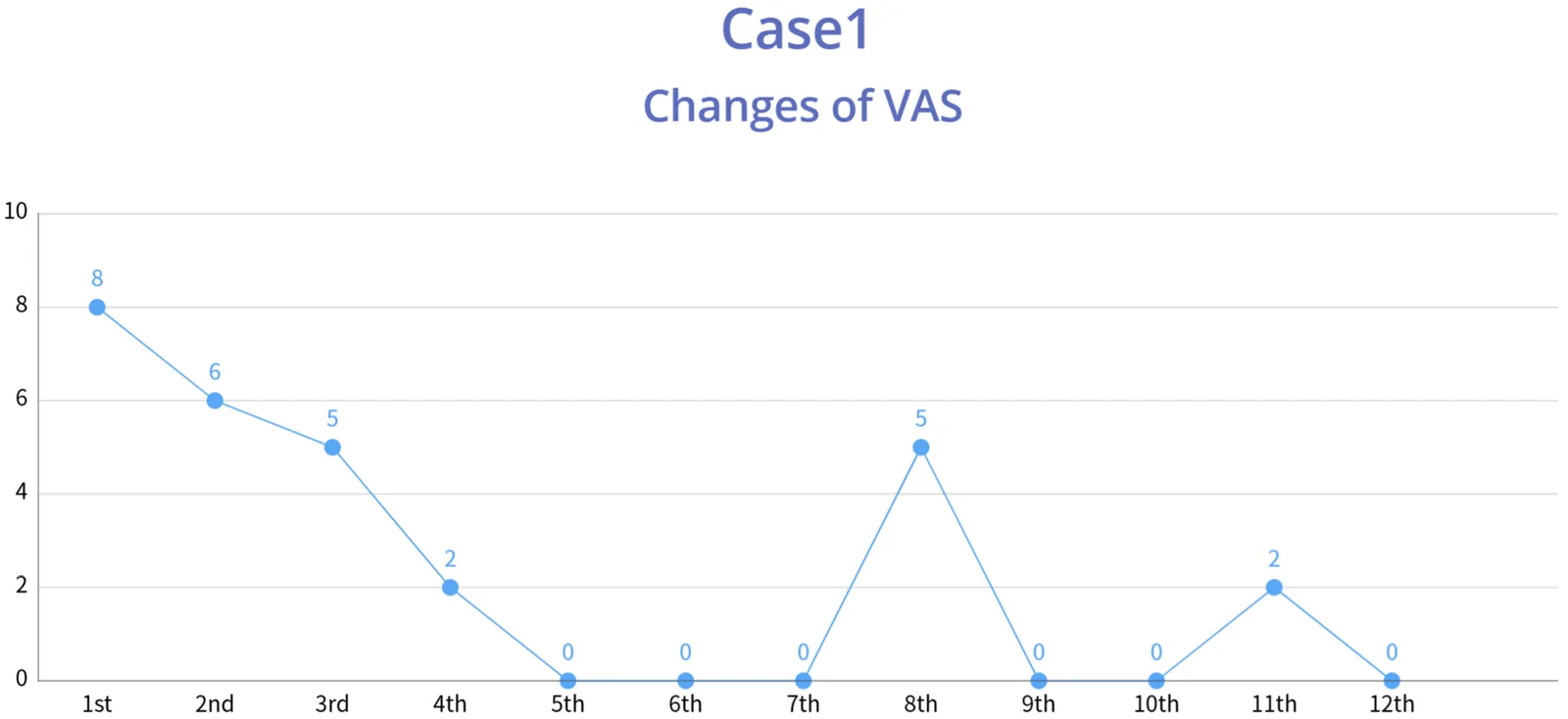
Change in VAS in case 1. VAS = visual analogue scale.

Quartz Chimsband was attached at the painful response sites (Fig. 3). At the 3rd visit, the patient reported that epistaxis was slightly improved. When he visited a 4th time, he stated that it was certainly relieved. At the 5th visit, epistaxis disappeared. However, at the 8th visit, the symptom recurred, and it was thought to be related to the patient’s fatigue because he had unusually early woken up for the golf trip the day before. We repeated treatment, and at the 9th visit on the next day it did not recur. Ten days later, it recurred and we repeated treatment. At the 11th and 12th visits, he stated that it did not recur. After 1 month, he visited again and reported that it had not recurred. The VAS evaluated by the patient decreased from 8 at the initial visit to 0 at the final visit (Fig. 4).

### 4.2. Case 2

#### 4.2.1. History and examination.

A 9-year-old female patient presented with epistaxis on both sides. The symptom had occurred 1 to 2 times a week and had lasted for 3 years. She had visited the hospital approximately 20 times a month and had been given laser therapy; however, she had felt no effect. Her present history included chronic fatigue, frequent eye blinking, and nasal congestion. She was a light eater and used to go to bed late (approximately midnight). Painful responses were exhibited at around LI20 and the sternocleidomastoid muscle TP on both sides.

#### 4.2.2. Treatment and progress.

**Figure 5.**
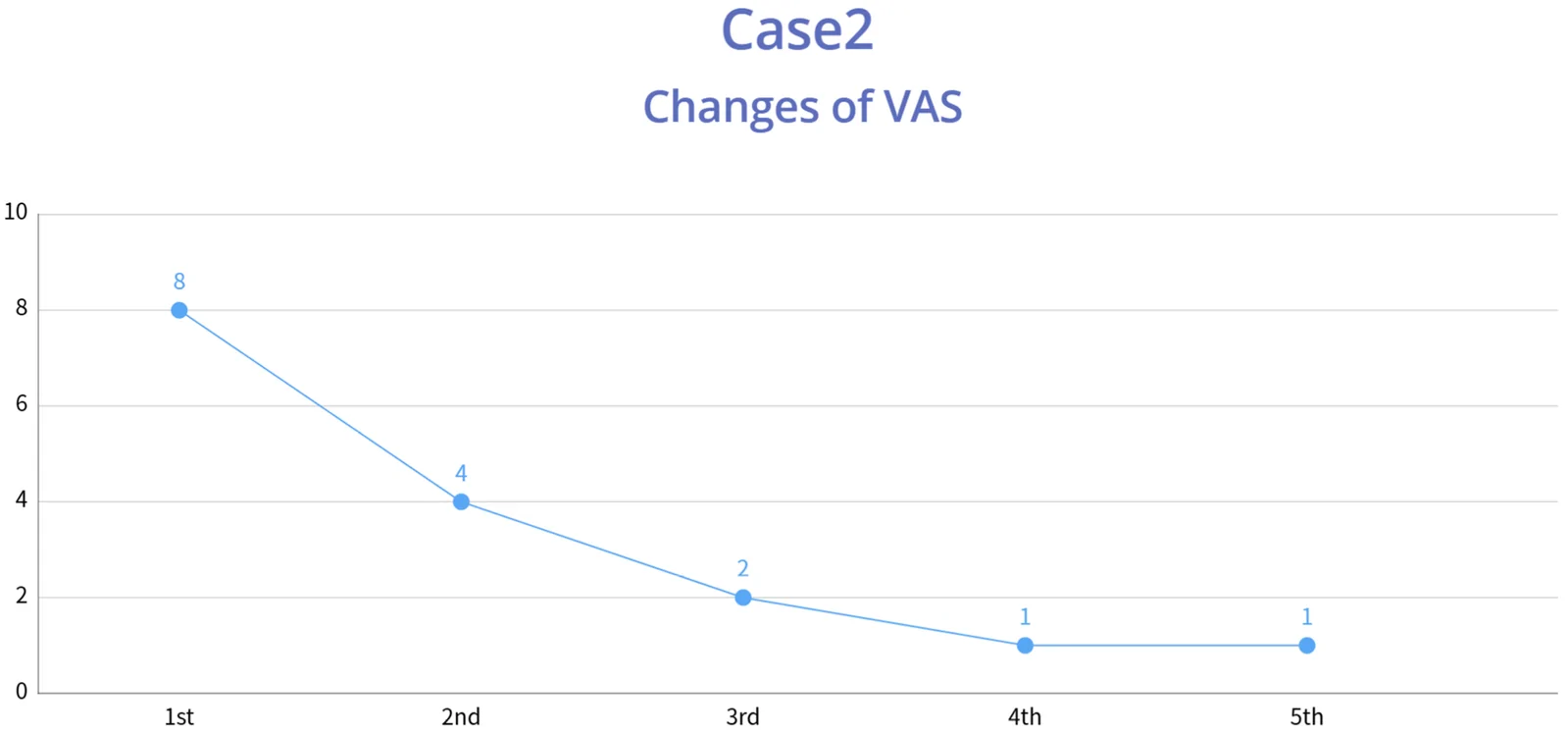
Change in VAS in case 2. VAS = visual analogue scale.

At the painful response sites, a quartz Chimsband was attached (Fig. 3), and immediately after attachment the patient felt ease in nose. At the 2nd visit, the patient reported that the nose bleeding stopped and the congestant got improved. At the 3rd visit, her parent stated that she rubbed nose much less than before, which was thought to be related to the congestant. At the 4th visit, her nose bleeding and congestant have already disappeared. At the 5th visit, she wanted to self-treat and was taught how to attach. One month later, she visited with abdominal discomfort and reported that the nose bleeding did not recur. VAS was reduced from 8 to 1 (Fig. 5).

## 5. Discussion

The patient in case 1 complained of epistaxis every morning that had lasted for 6 months. He had been treated in an ear, nose, and throat clinic using laser therapy, without improvement. After 3 sessions of our treatment, his symptom began to improve, and following the 4th treatment, the epistaxis stopped. However, at the eighth visit, the patient reported that the symptom recurred, and we thought that it might be due to fatigue. He woke up unusually early in the morning the day before, and hence the fatigue probably deteriorated, inducing injury of the mucosal capillary in the nasal sinus. After amelioration, the symptom recurred again at the 10th visit, and it seemed also to result from fatigue. In Eastern Asian traditional medicine, epistaxis is known to be induced by *Rising Heat* due to blood heat associated with fatigue.^[20]^ Therefore, the reason why this symptom had lasted for 6 months was thought to be that the patient usually overworked, and the heat used to rise easily, finally resulting in the injury of the nasal mucosa and capillary. Following 12 treatments, the symptom did not recur for 1 month, and it seems to be because the weakened nasal mucosa and capillary were recovered and the blood circulation in the nose was normalized owing to our taping therapy.

In case 2, a 9-year-old patient had suffered from epistaxis lasting 3 years and was comorbid for chronic fatigue, frequent eye blinking, and nasal congestion. Likewise, laser therapy of Western medicine was not effective. Based on the accompanying symptoms, we speculated that stiffness of the sternocleidomastoid muscle would be serious and widespread. In addition, the regimen of late sleeping that resulted in insufficient sleep time must have led to low sleep quality and accumulation of fatigue. This could contribute to the development of the TP of the sternocleidomastoid muscle. On the initial visit, attachment of the quartz Chimsband at around LI20, ST2, ST3, and the TP of the sternocleidomastoid muscle produced an immediate effect of feeling at ease. This result seems to have been produced because our taping ameliorated the nasal mucosa and capillary, perhaps by reducing possible inflammation. Five sessions of our treatment resolved the 3-year epistaxis, as the symptom did not recur during 1 month since the last treatment. Therefore, it seems that the quartz Chimsband is certainly effective for chronic epistaxis.

The sternocleidomastoid muscle has been known to be important for the treatment of ear, nose, and throat diseases and used to be treated with diverse modalities including acupuncture, physical therapy, and hyperthermia.^[10,21–23]^ Also, according to the meridian theory of Eastern Asian traditional medicine, the acupoints of LI20 (迎香), ST2 (四白), and ST3 (巨髎), corresponding to the surface of the paranasal sinus and the maxillary sinus, belong to the Yang Brightness (陽明) which controls the anterior face. Therefore, chronic nasal problems such as inflammation in the paranasal sinus and maxillary sinus could be easily perceived at these acupoints^[11,24]^ and we thought that resolving the painful response at these acupoints would be helpful to treat the epistaxis.

According to the myofascial pain syndrome theory, under a disease condition the related muscle’s TP is activated with being accompanied by the accumulation of pyruvic acid, the increased local temperature, the sensitive or painful response to palpation (finger pressure or pinch examination), and the abnormal bioelectric current.^[21]^ Also, it was suggested that the Qi energy, target of the acupuncture needle, gathering at the acupoint is the bioelectricity and hence it is important to utilize the electronic feature of the acupoint for a better acupuncture effect.^[25]^ Under a disease, the entity tries to self-treat using the injury current which appears at the local and related sites and using this injury current is effective to treat the disease.^[13]^ Therefore, regulation of the bioelectric current with our taping therapy at local and the related acupoints and TPs could ameliorate the flow of Qi (bioelectric current) in the related meridian and produce therapeutic effects.^[15–17]^ And here, we upgraded our tape, replacing the silver and the optic fiber with the quartz to regulate the bio-photon.

Until recently, transcutaneous electrical nerve stimulation and interferential current therapy have prominently been used as non-pharmacological therapies using bioelectric current to treat painful responses. Here, we utilized the bio-photon, the natural electromagnetic wave shown at painful response sites, i.e., disease-related TPs and acupoints, with quartz.

Quartz, a mineral described in Dong-Ui-Bo-Gam,^[18]^ is transparent and has excellent light penetrability. Therefore, we anticipated that utilizing its light utility would be beneficial for taping therapy. We substituted silver and optic fiber with quartz to better resolve the painful response at the related acupoints and TPs. If reactive oxygen appears at the painful response sites under a disease condition, the electromagnetic wave, so-called bio-photon, occurs, accompanied by inflammation, injury current, and pain.^[12]^ Therefore, we thought that enhancing self-treatment by utilizing (reflecting) this bio-photon with a medical tape containing quartz would be helpful in treating diseases.

On the other hand, it would be helpful for a better understanding of the mechanism if these therapeutic effects of the quartz Chimsband are confirmed through biochemical parameters such as the antioxidant^[26]^ and short-chain fatty acids.

Taken together, we found that attaching medical tape at the sternocleidomastoid muscle and around the acupoints of LI20, ST2, and ST3 after confirming the painful response is effective for the treatment of chronic epistaxis. Attaching quartz Chimsband, which reflects the bio-photon at the painful response site, can be considered an effective therapy. Given the benefits of taping therapy, this is not a painful therapy, and it might require a shorter treatment period than acupuncture because it provides persistent and long-lasting stimulation. In addition, it does not inject a material and just uses the bio-photon, which naturally occurs in the patient’s body therefore, it is rarely harmful and can be performed to the patients who are difficult to take invasive therapies including even gravida. Also, another benefit of quartz Chimsband is that the effect appears very quickly (immediately after attachment) because it works by regulating the bio-photon. Whereas, it should be noted that one weak point is that it perhaps induces a skin problem due to being sticky. Further studies should follow to investigate if quartz Chimsband could be effective for other diseases.

## 6. Conclusion

### 6.1. From this case study, we concluded the following:

1. Finding a painful or sensitive response at the related acupoints and/or muscle TPs can be useful for the treatment (taping therapy) of refractory epistaxis.
2. When painful responses are found at the sternocleidomastoid muscle and around the acupoints LI20, ST2, and ST3, removing the painful responses with taping therapy can be considered an effective therapeutic for epistaxis.
3. The taping therapy of Quartz Chimsband, reflecting the bio-photon at the related painful response sites, can be a useful therapeutic option for chronic epistaxis.

## Acknowledgments

This work was supported by the National Research Foundation of Korea (NRF) grant funded by the Korea government (RS-2025-00563810).

## Author contributions

**Conceptualization:** Hwa Soo Hwang, Bong Hyo Lee.

**Data curation:** Hwa Soo Hwang.

**Formal analysis:** Hwa Soo Hwang.

**Funding acquisition:** Bong Hyo Lee.

**Investigation:** Hwa Soo Hwang.

**Methodology:** Hwa Soo Hwang.

**Resources:** Hwa Soo Hwang.

**Supervision:** Bong Hyo Lee.

**Writing – original draft:** Hwa Soo Hwang, Bong Hyo Lee.

**Writing – review & editing:** Bong Hyo Lee.
